# Supplementary material for: Incidence, Etiology, and Risk Factors of Clinical Mastitis in Dairy Cows under Semi-Tropical Circumstances in Chattogram, Bangladesh
Source: Animals (Basel). 2021 Jul 30;11(8):2255. doi: 10.3390/ani11082255 (PMC8388477; doi:10.3390/ani11082255)
Supplement: Supplementary file 1 [file animals-11-02255-s001.zip › animals-1222325-supplementary.pdf]

Table S1. Descriptive analysis of farmer's perception towards mastitis and treatment strategies for clinical mastitis

Variables	Categories	Frequency (%)
What do you think about CM? (n[1]=30)	Unhygienic condition causes mastitis	9 (30)
	Milking fault causes mastitis	9 (30)
	Management fault causes mastitis	3 (10)
	This is a bacterial infection	3 (10)
	This results in udder swelling	2 (6.7)
	This is a water borne infection	1 (3.3)
	High yielding cows are repeatedly affected	1 (3.3)
	This results in milk color change	1 (3.3)
	Mysterious thing	1 (3.3)
What are the commonly used antibiotics? (n[1]=91)	Gentamycin	20 (22.0)
	Ceftriaxone	17 (18.7)
	Amoxycillin	11 (12.1)
	Colistin	6 (6.6)
	Kanamycin	5 (5.5)
	Neomycin	5 (5.5)
	Ciprofloxacin	4 (4.4)
	Oxy-tetracycline	4 (4.4)
	Streptomycin-Penicillin	4 (4.4)
	Ampicillin	3 (3.3)
	Amoxicillin-Cloxacillin	2 (2.2)
	Gentamycin intra-mammary tube	2 (2.2)
	Penicillin	2 (2.2)
	Sulfamethoxazole-trimethoprim	2 (2.2)
	Ampicillin-Gentamycin	1 (1.1)
	Ceftiofur	1 (1.1)
	Marbofloxacin	1 (1.1)
	Sulfadimidine-Trimethoprim	1 (1.1)
What are the supporting drugs used? (n[1]=28)	Probiotics	15 (53.6)
	No supportive drugs	7 (25.0)
	NSAID	2 (7.1)
	Homeopathic	1 (3.6)
	Antihistamines	1 (3.6)
	Dexamethasone	1 (3.6)
	Vitamin B-complex	1 (3.6)
Who prescribes the drugs? (n[1]=24)	Self then doctor	11 (45.8)
	Self	9 (37.5)
	Veterinarian	3 (12.5)
	Animal husbandry officer	1 (4.2)
What are the traditional treatments used? (n[1]=34)	Phytolocca (Homeopathic drug)	11 (32.4)
	Ice	4 (11.8)
	No traditional treatment	3 (8.8)
	Napthalene	3 (8.8)
	Cold water	2 (5.9)

Egg yolk emulsion	2 (5.9)
Belladona (Homeopathic drug)	2 (5.9)
Calcareasulf (Homeopathic drug)	2 (5.9)
Walking	1 (2.9)
Heat	1 (2.9)
Turmeric	1 (2.9)
Coconut oil	1 (2.9)
Aurum mureacum (Homeopathic drug)	1 (2.9)

%: Percentages; NSAID: Non-steroidal anti-inflammatory drugs n[1]=Total number of replies from 24 farms against each question. If one farmers mentioned multiple answers to a question, the replies were listed and counted inthe distinguished category under each question.
